# Noble Metals and Soft Bio-Inspired Nanoparticles in Retinal Diseases Treatment: A Perspective

**DOI:** 10.3390/cells9030679

**Published:** 2020-03-10

**Authors:** Valeria De Matteis, Loris Rizzello

**Affiliations:** 1 Department of Mathematics and Physics “Ennio De Giorgi”, University of Salento, Via Arnesano, 73100 Lecce, Italy; 2 Department of Chemistry, University College London, 20 Gordon Street, London WC1H 0AJ, UK; lrizzello@ibecbarcelona.eu; 3 Institute for Bioengineering of Catalonia (IBEC), The Barcelona Institute of Science and Technology, Baldiri Reixac 10-12, 08028 Barcelona, Spain; 4 Department of Pharmaceutical Sciences, University of Milan, via Mangiagalli 25, 20133 Milano, Italy

**Keywords:** retinal diseases, noble metals NPs, bio-inspired NPs, drug delivery

## Abstract

We are witnessing an exponential increase in the use of different nanomaterials in a plethora of biomedical fields. We are all aware of how nanoparticles (NPs) have influenced and revolutionized the way we supply drugs or how to use them as therapeutic agents thanks to their tunable physico-chemical properties. However, there is still a niche of applications where NP have not yet been widely explored. This is the field of ocular delivery and NP-based therapy, which characterizes the topic of the current review. In particular, many efforts are being made to develop nanosystems capable of reaching deeper sections of the eye such as the retina. Particular attention will be given here to noble metal (gold and silver), and to polymeric nanoparticles, systems consisting of lipid bilayers such as liposomes or vesicles based on nonionic surfactant. We will report here the most relevant literature on the use of different types of NPs for an efficient delivery of drugs and bio-macromolecules to the eyes or as active therapeutic tools.

## 1. Introduction

**Figure 1:**
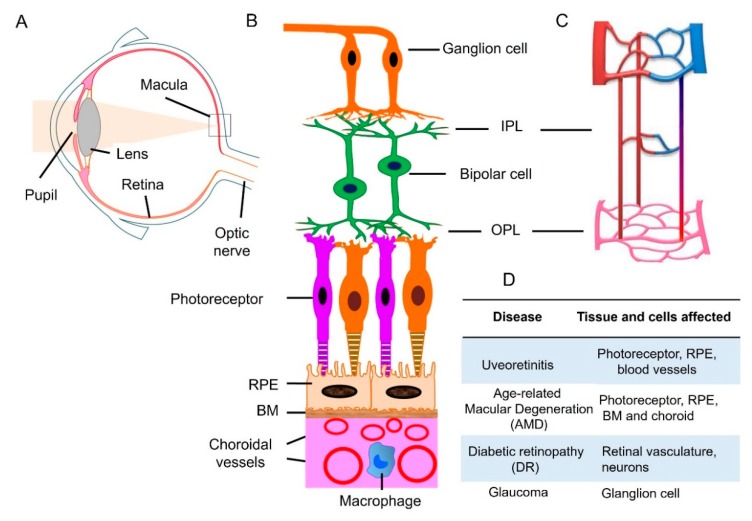
Retinal neuronal and vascular structure and retinal disease. (**A**) Diagram of a human eye. Light passes through the pupil and is focused by lens onto macula of the retinal layer at the back of the eye. (**B**) Retina consists of three layers of neurons, photoreceptor, bipolar, and ganglion cells. The retinal pigment epithelial (RPE) monolayer together with Bruch’s membrane (BM) form outer blood retinal barrier that separates the neuroretina from the choroid. Choroidal circulation provides oxygen and nutrients to outer retina. (**C**) The retina has an interconnected network of three vascular layers located in the ganglion cell/nerve fiber layer, inner plexiform layer (IPL), and outer plexiform layer (OPL). (**D**) retinal tissue and cells that are affected under different disease conditions. Adapted from reference [17].

The human eye is a unique and fragile structure prone to different types of pathologies. It is characterized by an anterior and the posterior segment [1]. Pupil, cornea, iris, ciliary body, aqueous humor and lens make up the anterior segment, whereas vitreous humor, macula, retina, choroid, and optic nerve are parts of the posterior segment [1]. The macula is a small area of the retina that contains photoreceptor, special light-sensitive cells, connected to a neuronal network [2]. Retina contains five types of neurons: photoreceptor, bipolar, ganglion, horizontal, and amacrine cells (Figure 1) [3,4]. The retina is the nerve layer that receives visual information, creating impulses that are transmitted through the optic nerve to the visual cortex in the brain [5]. Two types of photoreceptor cells exist: the rods and the cones. It is estimated that there are approximately 120 million rods and about six million cones in the human eye. The unique properties of photoreceptor cells expose them to a variety of genetic and environmental threats. Any dysfunction or deterioration of vital ocular tissue has a dramatic impact on a patient’s quality of life. Therefore, the retina is susceptible to several diseases that result in visual loss and blindness. As countries become wealthier and the per capita income increases, prevalence of blindness decreases, and causes of blindness change. In a poor African country, the major of blinding conditions are likely to be cataract and corneal scar. In the middle-income countries such as Latin America, the leading causes of blindness are glaucoma and diabetic retinopathy. Because cataract surgery is more readily available, fewer people become blind from cataract [6]. In wealthy countries, glaucoma and cataract continue to be very common and important conditions, but most of the blindness is due to retinal degenerative diseases (RDDs) [7], an heterogeneous group of phatologies characterized by progressive death of photoreceptors. RDDs can be caused by defects in proteins involved in photo transduction, synaptic transmission, retinal pigment epithelial (RPE) integrity function, intracellular trafficking and cilia function. It can be also related to toxic accumulation of retinoids. Each of these RDDs can lead to visual loss or complete blindness [8].

The most common RDDs include age-related macular degeneration (AMD), diabetic retinopathy (DR), and retinitis pigmentosa (RP). Cytomegalovirus (CMV) retinitis, proliferative vitreoretinopathy, Stargardt disease, and retinoblastoma can also occur and are usually associated with toxic retinoid biogenesis and accumulation [9]. Retinal diseases are already the most common cause of childhood blindness [10] with an incidence of 1.5 million people suffering worldwide. RP is characterized by a progressive loss of photoreceptors and its specific mechanisms are not fully understood. Following the loss of photoreceptor functionality, a retinal remodeling phenomenon is triggered as the final step, which then culminates with cell death and topological restructuring of the retina. The progression of retinal remodeling is similar to negative plasticity that occurs in some pathologies like trauma and epilepsy constituting substantial impediments to rescue strategies of all types [11]. Another process that involve the retina segment is neovascularization, a pathological phenomenon that provokes vision loss causing blindness due to its correlation with diabetic retinopathy and AMD [12]. In the neovascularization phenomenon, endothelial growth factor (VEGF) pathway has a key role [13,14].

Ocular diseases, and retinal diseases in particular, are still approached using rather invasive treatments, which are also not completely effective. The development of efficacious therapeutics is thus a primary research goal. So far, many delivery strategies have been explored, and the progress in the field of molecular genetics has led to the identification of genes involved in retinal disruption. For this reason, gene delivery systems represent one of the most promising approaches [15,16]. Delivery approaches can be broadly classified in two groups, whether they are based on a viral or non-viral method of delivery. Each system comes with advantages and disadvantages. For instance, although Adeno-Associated Virus (AAV) based vectors have high gene transduction efficiency, they face a major obstacle in terms of biosafety for clinical applications. The development of effective non-viral approaches for ocular disease is thus crucial. However, how to adequately deliver therapeutic agents to the back of eye (i.e., retina) remains a challenge. With the development of nanotechnology and bioengineering science, a wide range of innovations in this area have been achieved. This holds the promise for developing more effective ocular drug delivery systems.

In this review, we carefully report the last studies in which noble metal and soft (bioinspired) NPs were applied as new delivery agents for treatment of ocular disease in vitro and in vivo. We also provide perspective on opportunities and challenges for future nanotechnology in retinal drug delivery and therapies.

## 2. Drug Ocular Administrations

**Figure 2:**
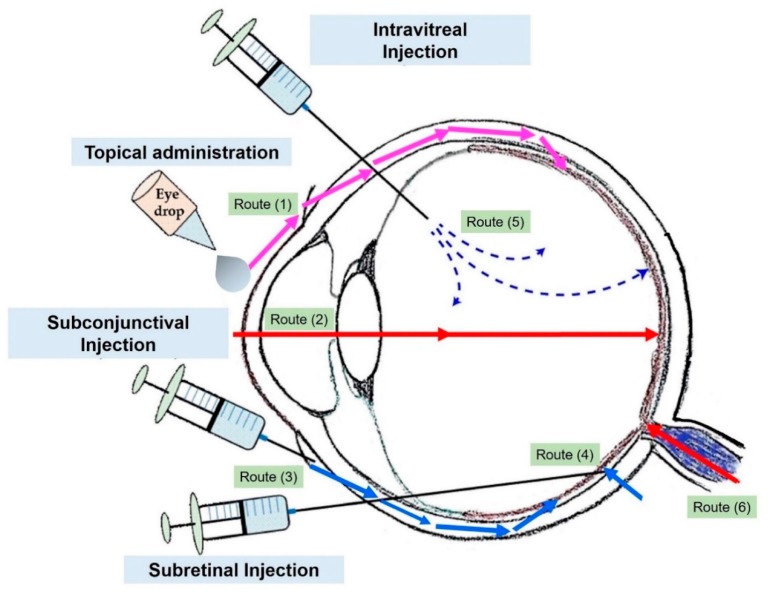
Common drug administration routes through the eye. Topical administration (**1**,**2**), subconjunctival injection (periocular route) (**3**), subretinal injection (**4**), and intravitreal injection (**5**). Adapted from reference [31].

The delivery of drugs in the eyes presents many difficulties. As in the case of the brain, it presents multiple physical boundaries: corneal and conjunctival epithelium, blood-aqueous barriers (BAB), and blood-retinal barriers (BRB). These barriers finely control the transit of molecules and fluids across eye and, in most cases, the drug is unable to enrich the deeper layers. The BRB consists of two different barriers: (i) the outer BRB characterized by endothelial cells lining the choroidal vasculature (fenestrated blood vessels) and by tight-junction-coupled RPE cells, and (ii) inner BRB. The latter is made by endothelial cells in conjunction with pericytes, astrocytes, and Müller glial cells. The inner BRB shows a lot of similarities with blood–brain barrier (BBB) with the exception of Müller glia [18]. In general, the most common drug administration is through eye drops and suspensions’ that target only the anterior segment of eye, mainly due to the patient compliance. With such a method, less than 5% of topically applied dose is delivered to deeper ocular tissues, such as in vitreous cavity [19]. The chemical nature of bioactive molecules also influences the uptake. For example, BRB is selectively permeable to lipophilic molecules in the presence of specific eye drugs transporters. In this way, the formulation of new therapies should take in consideration this limitation [20]. Ocular transporters are divided in two groups: the solute carrier (SLC) family and the ATP-binding cassette (ABC) family. The electrochemical gradient was used by SLC transporters to induce the uptake of molecules through cell membrane, whereas ABC transporters employ ATP [21]. However, physiological eye phenomena, such as tear turnover, reflex blinking, ocular static, or lacrimal drainage reduce the action of the drug and consequently the eye drop administration fails to treat retina disease [22]. As a matter of fact, in unstimulated conditions, total aqueous tear volume is 7 µL and the normal tear turn-over is about 1.2 µL/min [23]. Thus, the precorneal drug half-life is about 3 minutes [24]. Therefore, treatment of posterior segment injuries still remains a challenge. For all the reasons explained above, medical research seeks alternative routes such as intravitreal injections. This method consists of direct administration into vitreus via *pars plana* using a needle. In this way, a high concentration of drug is guaranteed at the retina level, but the molecule half-life depends on its molecular weight. In fact, proteins and peptides characterized by high molecular weight (ranging from 40 kDa to 70 kDa) and steric hindrance showed longer retention [25,26]. However, a lot of complications (hemorrhages, retinal detachment, cataracts) can manifest after injection [27]. An alternative method is periocular route (Figure 2) that was demonstrated to be an effective route to direct drugs to the posterior eye segment consisting of subconjunctival, sub-tenon, retrobulbar, peribulbar, and posterior juxtascleral [28]. Administration of drug by sub-tenon injection was found to be more suitable as demonstrated by Ghate et al. [29], where sodium fluorescein was used in rabbits’ eyes by subocular administration. The study concluded that injection of drug via sub-tenon resulted in highest vitreous concentration of sodium fluorescein (two sodium fluorescein (NaF) concentrations, 2.5 mg in 0.1 mL (c1) and 2.5 mg in 0.5 mL (c2)) compared to the other routes. However, also in this case, a lot of adverse effects have been observed, such as strabismus, hyphema, and intraocular pressure [30].

## 3. Physico-Chemical Properties of Nanomaterials

A “Nanomaterial” is defined as a “material with any external dimension in the nanoscale or having internal structure or surface structure in the nanoscale” (ISO, 2010) [32]. Similarly, a definition of “nanoparticle” is a “nano-object with all three external dimensions in the nanoscale” where nanoscale was defined as size ranging from 1 nm to 100 nm (ISO, 2008) [33]. NPs can be obtained by many synthetic routes, starting from chemical elements such as carbon, metals, metal oxide, biological molecules, and polymers [34]. The biological effects are strongly affected by physico-chemical properties of NPs such as surface charge, size, shape, and solubility. In addition, they exhibit greater surface area per unit mass compared to bulk materials [35]. Because of the above mentioned properties and of the material they are made, NPs are ideal tools to treat retinal disease as active components of the therapy without the help of drugs [36,37]. In addition, NPs with a smaller size (<20 nm) are demonstrated to have the ability to cross eye barriers including cornea, conjunctiva, and BRB [38,39]. NPs for ocular therapy include inorganic NPs (metal oxide and noble metal NPs), as well as soft-biopolymer-based NPs. Metal NPs are more suitable as active therapy tools (for their intrinsic properties), and soft-NPs are more efficient in encapsulating drugs and macromolecules due to their ability to form aqueous-suspended vesicles having a hollow lumen. All these nanomaterials are different in charge, shape, and size, but they are able to be internalized by cells to treat retinal disease [40]. Finally, recent applications of soft nanorobot (with size range in the molecular scale) have been studied. These structures have the capability to deliver active biomolecules in ocular sections due to their ability to make changes in a controlled and predictable manner to the environment following external stimuli [41].

### 3.1. Noble Metal NPs: The Case of Gold (Au) and Silver (Ag)

Noble metals, especially AuNPs and AgNPs, are characterized by unique optical properties. For this reason they are used for a variety of applications owing to collective oscillations of conduction electrons coupled with incident light [42,43,44,45]. This phenomenon, known as Localized Surface Plasmon Resonance (LSPR) is strongly influenced by NPs’ shape and the metal they are formed. The surface plasmon resonance bands of metal NPs can be tuned from visible to Near Infrared Region (NIR), which is a typical wavelength to penetrate and analyze biological tissues [46]. Even if AuNPs are the most studied in nanomedicine field due to their good chemical stability and well-controlled size/surface functionalization and biocompatibility, AgNPs have the advantage of possessing an antibacterial and antiangiogenesis effect [47,48].

### 3.2. Au and AgNPs: Safety Studies in In Vitro and In Vivo Retinal Models

#### 3.2.1. AuNPs

The use of novel nanomaterials necessitates a better understanding of potential adverse effects on biological entities [49]. These effects are strictly dependent on physico-chemical properties of NPs and evaluation of cytotoxicity is critical, especially from a medical field application perspective [50]. A lot of studies were conducted to assess biocompatibility in order to ensure safe drug release with low toxicity. In general, high degree of biocompatibility is verified when NPs interact with the living organisms without triggering unacceptable toxic, thrombogenic, carcinogenic, and immunogenic responses [51].

The unique physico-chemical properties of AuNPs make them a powerful tool in the biomedical field. They are widely used in photothermal treatment of cancer and in drug and gene delivery. For their synthesis, auric salts such as HAuCl_4_ are used as starting materials to obtain stable AuNPs with a size ranging from 1–100 nm (Brust or Turkevish [52,53]). In recent years, green syntheses of AuNPs has also been developed to reduce solvents and reduce/cap agents’ toxicity so that AuNPs can be synthetized from plant extracts or biomass [54,55]. AuNPs are easy to functionalize with a wide range of ligands ranging to polymer, DNA, peptides, RNA, and fluorescent molecules particularly suitable for bio-imaging applications [56]. Furthermore, various cytotoxicity studies demonstrated their low toxicity due to the inert nature of Au [57,58].

Regarding the biodistribution, a recent work demonstrated a rapid elimination of colloidal AuNPs from blood circulation in a rats model, and liver and spleen accumulation without inducing adverse effects or inflammation [59]. Au comprised in passion fruit-like nanoarchitectures (NAs) were studied in murine models demonstrating the excretion by renal and biliary pathways without nephrotoxicity up to 150 mg kg^−1^ [60]. Some studies reported pro-inflammatory cytokines expressions induced by AuNPs in different organs of rats [61] and mice [62]. However, the cytotoxic effects were temporary and normalized in a short time. The biocompatibility of Au is strictly correlated to their size, shape, concentration, as well as surface area as demonstrated by Biswas’s group [63]. They investigated the effects of Au spheres (5, 10, 20, 30, 50, and 100 nm), cubes (50 nm), and rods (10 × 90 nm) in cells in adult retinal pigment epithelial cell line 19 (ARPE-19). The cubes with an average diameter of 50 nm and spheres (50–100 nm) resulted in being biocompatible up to 750 μg of concentration. NPs with a smaller size (<30 nm) and nanorods (10 nm) showed higher toxicity, but they are more able to be uptaked by cells. Kim et al. [64] used C57BL/6 mice to demonstrate the ability of AuNPs (20 nm) to cross BRB and enriched retinal layers after intravenous administration. NPs greatly accumulated in neurons (75 ± 5%), followed by endothelial cells (17 ± 6%) and peri-endothelial glial cells (8 ± 3%) without affecting both viability of endothelial cells, astrocytes, retinoblastoma cells, and expression of functional biomolecules. After five weeks of post administration, the thickness of retinal layers was undisturbed without any damage. Bakri et al. [65] used Dutch-belted rabbits to test biocompatibility of AuNPs. They used two groups of rabbits receiving a 67 μmol/0.1 mL and 670 μmol/0.1 mL of AuNPs into one eye respectively by intravitreal injection. After one month, evident inflammation or adverse effects were not detected also at a high dosage. Soderstjerna et al. [66] used AuNPs and AgNPs with 19 and 89 nm citrate capped to test biocompatibility in in vitro cultured retinas using 800 NPs/cell. The retina tissue model offered a sustained long-term retinal organization and controlled serum-free conditions. This study demonstrated that low concentrations of Au and AgNPs have adverse effects on retina and can induce apoptosis and oxidative stress. A strong connection between NPs hydrodynamic diameter, surface functionalization/concentration and the biological effects is evident. In particular, the authors showed that NPs size should be large enough to avoid their fast flow into circulation, but small enough to be administrated and easy cross the BRB [67].

Starting from this assumption, the application of AuNPs is influenced by these parameters that should be taken in consideration to customize nanotools for a specific biomedical application.

#### 3.2.2. AgNPs

Numerous in vitro and in vivo studies demonstrated the toxicity of AgNPs that perturbs several cellular pathways [4]. The most accepted mechanism through which they induce side effects, despite still under investigation, seems to be related to Ag ions released from NPs surface [68,69,70]. In terms of production process, AgNPs can be synthetized by different chemical routes such as chemical-based reduction [71], sol-gel, or green synthesis [72,73].

Jun et al. [74] used zebrafish embryos as model organism to test their delivery at an ocular level. They investigated the toxicity of AgNPs at concentration of 0.4 mg/L and observed down- expressions of different lens crystalline genes by unknown mechanisms that either induced apoptosis or blocked nuclear DNA or RNA export.

Sriram et al. [75] demonstrated the cytotoxicity and Reactive Oxygen Species (ROS)-induced apoptosis after exposure of AgNPs with two different size (22.4 and 42.5 nm) in bovine retinal endothelial cells (BRECs) finding size-dependent toxicity. As a matter of fact, smaller NPs induced adverse effects on cells. Kim and colleagues [76] exposed New Zealand white rabbits (according to OECD test guidelines) to AgNPs in order to understand the possible eye irritation effect. Oedema and conjunctival redness have been observed after 1 h of AgNPs incubation, but the cornea, iris, or conjunctiva resulted unaffected after 24, 48, and 72 h of NPs exposure. In similar experiments, the instillation of AgNPs (5000 ppm) were tested on another in vivo model, the guinea pigs, observing a transient conjunctivae irritation during 24 h of early observation [77].

## 4. Noble-Metal NPs as Therapy and Diagnostic for Retinal Disease

**Figure 3:**
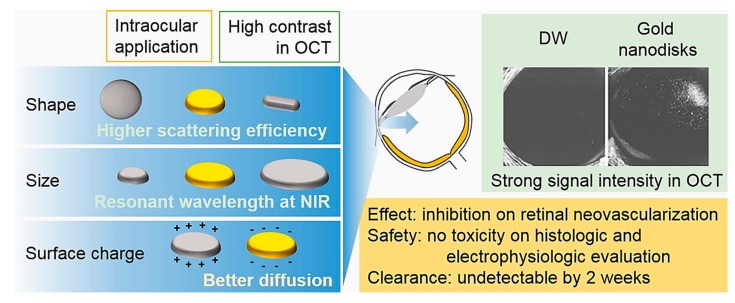
Schematic representation of gold nanodisks (GNDs) effects in retinal degeneration and Optical Coherence Tomography (OCT) imaging. The GNDs were particularly suitable for their optical properties connected to the shape: GNDs absorbed in the Near Infrared Region (NIR). The size and surface charge also influenced the eye diffusion. In OCT images, GNDs exhibited a strong signal compared to the dual window (DW) processing method, which is used to detect modulation of OCT signals due to scattering or absorption. Reprinted from [80] Copyright (2017), with permission from Elsevier.

AuNPs scatter or absorb light at specific wavelengths as a function of their physico-chemical properties. This characteristic is particularly suitable for bioimaging and to treat some diseases like cancer by NIR-triggered photothermal therapy (PTT), in which light is converted to heat in the tumor site where NPs accumulate [78]. In the NIR (650–900 nm), the absorption coefficients of haemoglobin and water are very low, and for this reason, the penetration of NIR wavelength in tissues is very high, allowing NPs stimulation without causing damage [79]. Song et al. [80] reported application of gold nanodisks (GNDs) on human retinal microvascular endothelial cells (HRMEC) and mouse oxygen-induced retinopathy model. The GNDs were optically suitable for nanomedicine applications due to their ability to produce detectable signals regardless of polarization or direction of light source and to scatter in NIR region (830 nm). In addition, GNDs were internalized less efficiently in cells with respect to AuNPs or Au rods (AuNRs), thus minimizing the ROS production and other several adverse effects. The authors used differently charged anisotropic NPs (nanorods or nanodisks) and compared them with AuNPs. After simulating the role of different sizes to select the best diameter (160 nm), they showed that GNDs were able to bind vascular endothelial growth factor (VEGF) inhibiting in vitro angiogenesis with high performance and abolished the VEGF-induced migration of endothelial cells. When these nanostructures were applied to mice eyes, a strong signal in optical coherence tomography (OCT) was detected with a consequent reduction of retinal degeneration (Figure 3). The OCT system involves the use of light energy that, once absorbed by tissues, causes a thermos-elastic expansion generating ultrasonic waves detected by a transducer.

Recent therapies to treat some diseases strictly connected to retinal degeneration consider the transplantation of photoreceptor precursors (PRPs) as efficient support improving visual acuity [81]. The analysis of cell survivor in the host retina is a key factor in this kind of treatment. Chemla et al. [82] used AuNPs in order to improve the tracking of transplanted PRPs. Cells were labelled with photothermic AuNPs and transplanted in the vitreous and sub-retinal space of Long-Evans pigmented rats (4–8 weeks old) by gauge needle.

By using coherence tomography, computed tomography and fluorescence fundus imaging, NPs have been tracked for 1 month without recording adverse effects. Real-time tracking is essential for evaluating the efficiency of retinal cell replacement therapies. These studies confirmed the possibility to use AuNPs to improve retinal cell therapy in humans. AuNPs-based photothermal therapy has been also used to treat eye tumors in vitro and in vivo. Some eye cancers provoke retinal detachment leading to a decreased visual eye activity.

In this framework, Kim et al. [83] tested anticancer properties of Doxorubicin (Dox)-conjugated Fucoidan (Fu)-encapsulated AuNPs (Dox-Fu@AuNPs) for the treatment of VX2 squamous carcinoma cells and xenograft tumors in New Zealand White rabbits. After intra-tumoral injection of Dox-Fu@AuNPs in the eye, a complete suppression of the tumor has been observed by dual-chemotherapy and PTT treatment. No damage to retina has been also detected. These composite nanostructures enabled the improvement of the OCT imaging technique (thanks to the selective light absorption of AuNPs) [84].

The quality of photoacoustic-based imaging was also improved thanks to selective light absorption of AuNPs. In this way, a double effect in terms of therapy and diagnostic was achieved.

As reported above, OCT is largely used for imaging in ocular clinical research due to the possibility to take structural information based on the reflectivity of tissue. In this respect, Gordon and colleagues [85] confirmed that Au nanorods (with a peak absorption of 750 nm and diameter of 10–35 nm) injected in the retina of Wild-type C57BL/6 mice are able to enhance the contrast, thus significantly improving the quality of a scanned lesion. Additionally, intravitreal injection of Au nanorods functionalized by anti-VEGF antibody triggered a reduction in the extent of anatomic laser damage and lesion-associated photothermal signal density in mice treated in laser induced choroidal neovascularization (LCNV) model and injected with ICAM2-targeted GNR. AuNPs (3–5 nm) were also used as inhibitor of VEGF on choroid-retina endothelial cells (RF/6A) [86]. A similar effect was showed using AgNPs, which have been found to reduce the VEGF activation and induced cell proliferation and migration in bovine retinal endothelial cells [87]. In addition, AgNPs were shown to inhibit the angiogenesis, typical of metastasising cells and of retinal neovascularization development. Ag is widely used because it has the great advantage of being a cost-effective material. For this reason, it is a good candidate for the replacement of the current therapies used for the treatment of various retinal angiogenic disorders. In this respect, Sheikpranbabu and co-workers [88] investigated the effect of green AgNPs, obtained from *Bacillus licheniformis*, on VEGF-and Interleukin 1 β (IL-1β) expression—that induced retinal endothelial cell permeability. AgNPs were able to stop VEGF-and IL-1β-induced permeability in retinal endothelial cells from porcine retina.

## 5. Bio-Inspired NPs for Ocular Drug Delivery

The use of bio-inspired materials mimicking natural components for applications in living organisms has widely spread recently [89]. In fact, inorganic NPs have the serious drawback of not being degradable and are also difficult to get excreted from the body [90]. Bio-inspired nanomaterials, including liposomes, niosomes, and chitosan/alginate NPs have been then explored as valid alternatives to metal NPs for treatment of several diseases [91]. In addition, these ‘soft’ NPs have been applied for drug delivery in the posterior segment of eye, including retinal photoreceptors.

### 5.1. Liposomes as Drug Delivery Carrier

Liposomes consist of a lipid bilayer, resembling a cellular membrane, that surrounds an aqueous core. These structures are widely used in pre-clinical studies to improve drug delivery, for example through Blood Brain Barrier (BBB) [92]. The BBB and BRB have the same functional properties [93,94]: so liposomes improve drug delivery to the retina and its photoreceptors due to their high half-life, permitting the long term drug absorption [95]. Liposomes also offer the ability to significantly improve pharmacokinetics and pharmacodynamics of any loaded drug compared to its free-circulating counterpart. They are also considered as highly biocompatible and biodegradable as well up to some extent both in vitro and in vivo. For this reason, several anticancer drugs such as doxorubicin, daunorubicin, and epirubicin were encapsulated in liposomes core with results demonstrating a significant decrease of many severe adverse effects (nausea, weakness, vomiting) with respect to the same concentration of free (non-encapsulated) drug. In this context, Zhang et al. [96] subcutaneously injected tacrolimus-encapsulated liposomes in Lewis rats with autoimmune uveoretinitis (EAU) by subcutaneous injection. Confocal analysis over rhodamine-conjugated liposomes (containing also the tacrolimus) revealed that such vesicles remained in ocular fluids even after 14 days. They found a reduced intraocular inflammation and decrease of EAU development without observing significant adverse effects.

Liposomes encapsulated with the anti-inflammatory and anti-angiogenic drugs berberine hydrochloride and chrysophanol were applied in myoblasts and in ischemic animal models. These drugs have the ability to contrast oxidative stress by enhancing the activities of antioxidant enzymes such as superoxide dismutase (SOD) and glutathione peroxidase (GSH-Px) [97,98]. Berberine hydrochloride and chrysophanol have been used in ocular liposome-mediated delivery to overcome their low stability. In this respect, a recent study [99] showed a liposome system that can entrap the two drugs at same times using the third polyamidoamine dendrimer (PAMAM G3.0) as a delivery carrier for age-related macular degeneration (AMD) therapy in the ocular posterior chamber.

**Figure 4:**
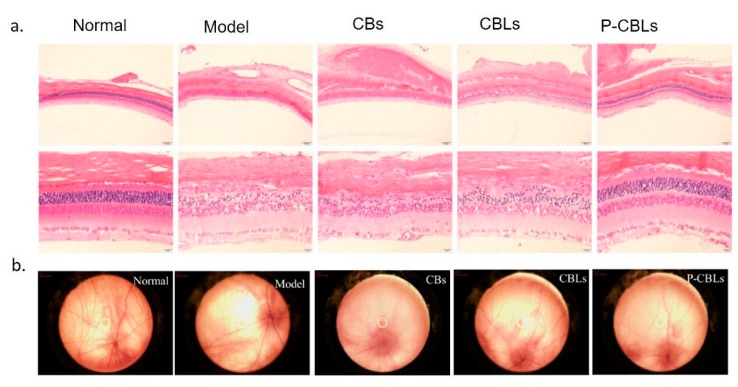
(**A**) Protection evaluation against photo-oxidative retinal damage in a light-damaged animal model by the use of different formulation: chrysophanol–berberine hydrochloride suspension (CBs), compound liposomes (CBLs), and PAMAM coated compound liposomes (P-CBLs). Retina was stained with hematoxilyn/eosin (scale bare = 20 µm). (**B**) Fundus retinography after incubation with the different formulations. Adapted from [99] under the terms of the Creative Commons Attribution 4.0 International License.

PAMAM G3.0-coated compound liposomes were used to evaluated HCECs (Human Corneal Epithelial Cells) internalization, trans-corneal permeability in New Zealand white rabbits (weighing 2.0–2.5 kg) and male Sprague–Dawley rats (weighing 210–250 g). Pharmacokinetics were evaluated together with in vitro assessments of anti-reactive oxygen species (ROS) efficacy. The PAMAM G3.0-coated compound liposomes enhanced bio-adhesion on rabbit corneal epithelium after topical administration and exhibited great permeability in HCECs cells. The possibility to deliver a protective agent against photo-oxidative retinal damage in a light-damaged animal model was also investigated through the use of dendrimers (Figure 4A). Results showed that dendrimers encapsulated with chrysophanol–berberine hydrochloride induced the greatest protection of retinal function in light-exposed rats compared to unloaded dendrimers and free drug. In addition, no inflammation and damage of cornea, iris, and conjunctiva was observed after drug-loaded dendrimers instillation for 14 days. Upon exploring the fundus of the eye (interior surface of eye opposite the lens including the retina, optic disc, macula, fovea, and posterior pole) [100], modifications in the vessel structure and distribution have not been observed after different treatments. (Figure 4B).

Liposomes offer also the great ability of an easy surface functionalization with a plethora of active macromolecules and targeting moieties that bind in a selective manner some specific receptors constitutive of different cell lines [101,102].

The surface modification of liposomes has also been widely studied to improve their specificity of targeting and to deliver them in different eye compartments. It has been reported [103] that the cationic PEGylated liposomes having an average zeta potentials below +20 mV were able to efficiently penetrate the murine retina. In contrast, liposome having surface zeta potential values above +20 mV were retained on vitreous section. This did not change whether PEGylated or non-PEGylated liposomes were used. Another critical factor for a liposome-based therapy is the phospholipids composition characterizing them. For example, edaravone-loaded egg L-phosphatidylcholine liposomes prevent retinal ganglion cells (RGCs) from undesired apoptotic processes [104]. This drug is a free radical scavenger used for acute brain infarction and amyotrophic lateral sclerosis treatment [105]. Surprisingly, minor RGC protection was observed with edaravone-loaded distearoyl phosphatidylcholine (DSPC) liposomes. This evidence suggest a link between the viscoelastic properties of the lipid bilayer and the final biological effect. In this specific case, the higher fluidity was a key factor to improve the ocular therapy. Another study [106] also tested the use of liposomes for the treatment of different kinds of hereditary degeneration of the retina. The degeneration of retina affects rod photoreceptors important for capturing light under low illumination conditions. Because of the pathology development, the patients affected by retina degeneration become aware of defects only when lesions are in an advanced stage. A key factor for this disease seems to be rods-specific Pde6b gene mutation in the transduction pathway that triggers an excessive accumulation of cGMP (that have two effectors: cyclic nucleotide gated ion channels (CNGCs) and cGMP-dependent protein kinase (PKG) [107]. Consequently, rod cell death was induced and in some cases the secondary loss of cones was observed [108]. The authors identified a liposomal cGMP analogue that was able to overcome the blood–retinal barrier (BRB) while strongly preserving retinal function and reducing rod photoreceptor loss in three different in vivo models for retina degeneration [106,109]. The vesicle structure of liposomes was suitable to confine proteins and peptides into aqueous core overcoming potential agglomeration and protein misfolding events. However, the surface functionalization with peptides is the most common method used for retina therapy. For example, liposomes functionalized with tanticoagulant annexin A5 have been demonstrated to have enhanced uptake levels through corneal epithelial barriers [110].

This vector has been injected topically to deliver 127 ng/g of bevacizumab to posterior segment of both rats and rabbit eye. Annexin A5-functionalized liposomes loaded with bevacizumab were also rapidly delivered to retina of the two animal models. Annexin A5 unilamellar liposomes made by different polymers (2-dipalmitoyl-sn-glycero-3-phosphocholine,1,2-dioleoyl-sn-glycero-3-phospho-l-serine and cholesterol) have been also used to target transforming growth factor (TGF)-ß1 into the vitreous of rabbits [111]. It has been found that a higher concentration of molecule with respect to the experimental group treated with the unloaded drug only. The choice for human recombinant TGF-β1 relied on the drug’s ability to prevent retinal damage elicited by Aβ oligomers.

### 5.2. Niosomes for Drug and Gene Delivery

Gene therapies is an approach where diseases are cured by modifying the expression of specific genes regulating pathology. This happens through the administration of nucleic acids aiming to replace abnormal gene sequence [112]. Liposomes and lipoplexes have been taken into consideration as suitable non-viral vectors platform for gene editing. Non-viral gene delivery approaches were widely applied in clinical trials since 2004 to attempt replacing the use of viral vectors that possess high risk of inducing oncogenesis, immunogenicity, mutagenicity phenomena [113].

Among non-viral vectors, niosomes have been recently tested as a potential candidate for gene delivery. Niosomes are vesicular systems comprising of bilayer made up of nonionic surfactants and they can internalize several types of bio-macromolecules such as nucleic acids and drugs. The most commonly used surfactant are Sorbitan fatty acid esters, Spans (Span 20, 40, 60, 65, 80, and 85), Tweens (20, 40, 60, and 80) [114] and brij (30, 35, 52, 58, 72, and 76) [115].

**Figure 5:**
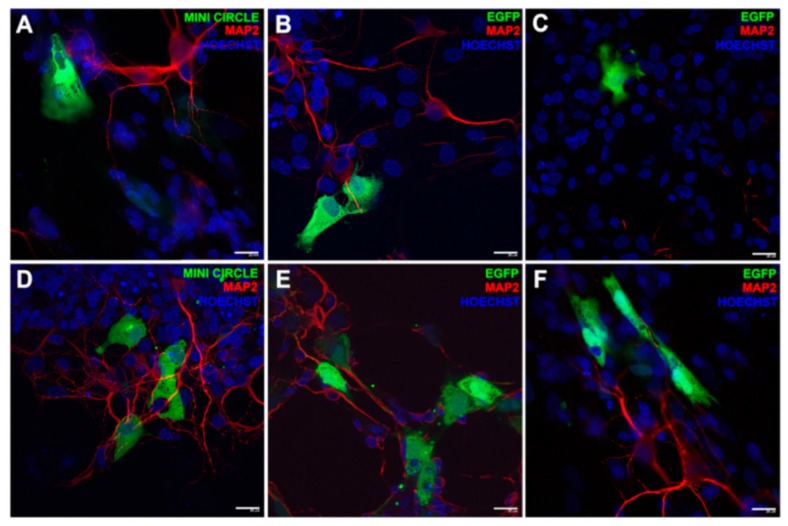
Fluorescence immonostaining by GFP expression in embrionary rat retinal primary cells. The green signal was referred to the transfection event of DST20 nioplexes with MC-GFP, (**B**) pGFP 3.5 kb, or (**C**) pEGFP 5.5 kb. (**D**–**F**) Positive controls incubated with Lipofectamine™ 2000. Nuclei were stained with Hoechst 33,342 (blue) and neuronal dendrites with MAP2 (red). Scale bars: 20 μm. Reprinted from [116] Copyright (2019), with permission from Elsevier.

In a recent study [116], niosomes made by cationic lipid 1,2-di-octadecenyl-3-trimethylammonium propane, Squalene, and Tween 20 have been combined with GFP-encoding genetic materials consisting on minicircle (MC-GFP, 2.3 kb), its parental plasmid (pGFP, 3.5 kb), and a larger plasmid (pEGFP, 5.5 kb). Their efficacy in retinal disease treatment has been investigated. These noisome-DNA complexes, named as nioplexes, demonstrated good transfection ability in human ARPE19 retinal pigment epithelium cells (in vitro) and in embrionary rat retinal primary cells (in vivo). Fluorescence showed GFP positive signal emission after transfection with the nioplexes carrying plasmidic DNA. In addition, such transfected cells showed physiological glial cell morphology confirming good cell viability in all tested cases. (Figure 5). The successive injections of nioplexes in rat retinas confirmed the higher capacity of cationic niosomes vectoring minicircle DNA to deliver genetic material into retina cells. Therefore, nioplexes based on cationic niosomes vectoring minicircle DNA are demonstrated as powerful agents in retinal diseases therapies. Cationic formulations made by a combination of Tween-60:DOTMA:lycopene have been used to transfect ARPE-19 cells with pCMS-EGFP plasmid with a transfection efficiency close to 35% [117]. Also in case, in vivo experiments in rat eye showed the efficiency of nioplexes to transfect outer segments of the retina, Niosomes constituted by Span 40 or Span 60 and cholesterol were produced to encapsulate and topically deliver in vivo acetazolamide. The intraocular pressure (IOP) after drug delivery system application was reduced in rabbits. In particular, multilamellar acetazolamide niosomes developed with Span 60 and cholesterol (7:4 molar ratio) were more efficient in reducing IOP, and at same time, no irritation and retinal damage were observed [118]. Gugleva et al. [119] prepared niosomes by different surfactants to deliver doxycycline hyclate in rats eye. Doxycycline hyclate is an antibiotic used for the treatment of eye infections. It was demonstrated to inhibit effect on matrix metalloprotease 9 (MMP-9) activity and mitogen activated protein kinase in experimental murine dry eye [120]. Niosomes were synthesized using several surfactants (Span 20, Span 60, Span 80, Tween 60) and cholesterol in different molar ratios and tested on male albino rabbits. The experiments clearly showed that the prepared niosomal formulations were high tolerated and did not induce any irritations.

### 5.3. Hydrogels-Based Nanocarriers

Hydrogels are three-dimensional, cross-linked networks of natural or synthetic water-soluble polymers that are suitable for applications like tissue engineering and controlled/sustained drugs delivery [121,122]. The aqueous environment is particularly advantageous to confine soluble active molecules, oligonucleotides, and proteins. For this reason, they are powerful tools in retinal treatment disease [123]. In addition, smart or environment-responsive hydrogels were synthetized to improve drug delivery efficacy [124].

#### 5.3.1. Natural Hydrogels: Chitosan NPs (CHNPs)

Chitosan is a natural hydrogel cationic polysaccharide of co-polymers glucosamine and N-acetyl glucosamine [125] obtained by alkaline deacetylation of chitin that is constitutive of crustacean shells of shrimps, lobster, and crab. It presents a positive charge that can be exploited to form polyelectrolyte vesicular complexes able to act as delivery systems. The positive charged chitosan is also ideal to bind a negatively charged corneal surface, to improve precorneal retention time and to decreases clearance [126]. In addition to chitosan, also the biocompatible alginate is extensively employed in biomedical applications. Alginate can also be a useful biopolymers for designing responsive hydrogels [127]. Because of its characteristics, alginate is also used for ophthalmic applications often in combination with chitosan [128]. For example, chitosan coated sodium alginate NPs [129] loaded with Fluorouracil (5-FU) have been successfully tested for eye delivery demonstrating a high 5-FU release in aqueous humor compared to free drug in rabbit eye. In another approach, chitosan NPs were encapsulated with levofloxacin for the treatment of ocular infection [130]. The formulation of NPs were optimized to enhance corneal retention without inducing eye irritation. These nanovectors demonstrated strong antibacterial agents against *Pseudomonas aeruginosa* and *Stafiloccocus aureus*.

Andrei and co-workers [131] designed biocompatible chitosan and alginate-based micro/NPs with a specific morphology aiming to deliver cefuroxim, a second-generation cephalosporin antibiotic. The authors performed an in vivo assessment to evaluate the biodistribution following an intravitreal administration of fluorescein-chitosan NPs on wistar rats. They observed an efficient NPs distribution in lens and retina. Similarly, chitosan-dextran NPs have been employed to deliver the antibiotic moxifloxacin that has been retained for a long time in vivo thanks to the mucoadhesive properties of NPs [132].

In recent work, humanin, a neuronal peptide that mediated inflammatory responses, was used in the treatment of age-related macular degeneration (AMD) [133]. This peptide was a suppressor of the IL-6 cytokine receptors and protects neuronal cells from necrosis [134]. The authors studied the effect on the inflammatory response reduction using chitosan NPs as vector vesicle (with a size of 346 ± 52 nm) in which humanin (a micropeptide encoded in the mitochondrial genome) derivative AGA-(CR8) HNG17 (AGA-HNG) were encapsulated. The effects of NPs were assessed in retinal pigment epithelial cells. The percentage release profile of free protein after five days was 60%, after which it enriched a plateau whereas the nanostructured exhibited a slow and substantial release with 80% of protein released after fifteen days. Cheng et al. [135] developed a thermosensitive hydrogel containing latanoprost and curcumin-loaded NPs (CUR-NPs). Curcumin was selected because of its antioxidative and antiinflammatory properties that made it suitable also for treatment of eye pathologies such as chronic anterior uveitis and light-induced retinal degeneration [136]. Latanoprost was chosen because it acted on IOP restore due to uveoscleral outflow increase [137]. In glaucoma disease, IOP is generated by loss of aqueous humor drainage through the uveoscleral or trabecular outflow pathway that can be damaged by oxidative stress at trabecular meshwork level [138]. In this condition, Latanoprost and CUR-NPs were demonstrated to decrease the oxidative stress in trabecular meshwork (TM) cells that resulted in decreased levels of apoptosis, inflammation-related gene expression, and mitochondrial damage [135]. In another work [139], neuroprotective and neuro regenerative agent Erythropoietin (EPO) was encapsulated in chitosan/hyaluronic acid (HA) nanoparticulate (size ≤300 nm). Different formulations have been tested and delivered to porcine corneas, scleras, and conjunctivas by ex vivo permeation, finding out a good combination between targeting efficiency and biocompatibility.

Pandit et al. [140] encapsulated high levels of bevacizumab (an anti-angiogenesis drug) within chitosan-coated PLGA NPs (size 222.28 ± 7.45) and demonstrated its efficient deliver to the retina followed by efficient release. As matter of fact, CS-coated NPs showed better permeability of the bevacizumab across sclera as compared to the free drug solution. Another category of carrier is made by glycol chitosan (GCS) NPs [141]. These biocompatible NPs, demonstrated to load high amount of plasmid DNA, have been successfully delivered to retinal pigment epithelium of adult wild-type albino mice. More importantly, the encapsulated pDNA was intact and maintained a proper conformation.

#### 5.3.2. Synthetic Hydrogel Based Nanocarriers

The synthetic hydrogels are easy to customize and indeed are a close model of biological matrices. Hydrogels exhibit high hydrophilicity and high hydration [142]. The most commonly used are poly(hydroxyethyl methacrylate) (PHEMA), poly(ethylene oxide) (PEO, also called poly(ethyleneglycol) (PEG)), polyvinylpyrrolidone (PVP), poly(acrylic acid)(PAA), polyacrylamide (PAM), and poly(vinyl alcohol) (PVA) [143]. These polymers are all used in several ophthalmic applications with good results [144,145,146,147].

Anti-VEGF antibodies (anibizumab or aflibercept) were loaded within poly(lactic-co-glycolic acid) microspheres, and successively suspended within poly(N-isopropylacrylamide)-based hydrogel and injected into a laser-induced rat model of choroidal neovascularization (CNV). Upon ocular administration, the rats showed smaller CNV lesions (less than 60%) with respect to non-treated animals [148].

Agrahari et al. [149] obtained novel pentablock (PB) copolymer (PB-1: PCL–PLA–PEG–PLA–PCL) based nanoformulations suspended in a thermosensitive gelling copolymer (PB-2: mPEG–PCL–PLA–PCL–PEGm) with a size of 150 nm. This composite nanoformulation was used to deliver macromolecules to slowly release drugs without toxic effects on the posterior segment of the eye. In vitro tests performed on ocular and mouse macrophage (RAW 264.7) demonstrated their biocompatibility and potential to be clinically translated.

**Figure 6:**
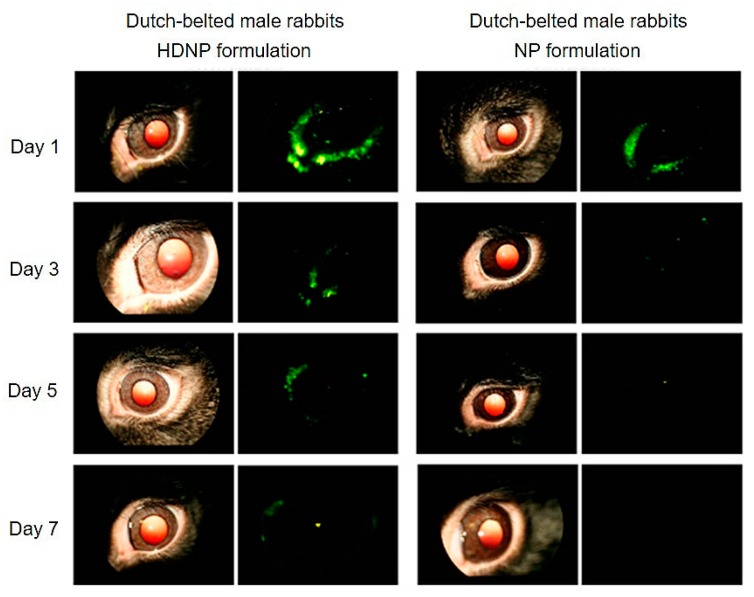
Fundus camera acquisitions of rabbits’ eyes after hybrid polyamidoamine (PAMAM) dendrimer hydrogel/poly(lacticco-glycolic acid) (PLGA) nanoparticle platform (HDNP) and NP Formulations (NP) topical administration FluoSpheres were confined in nanosistems to follow the distribution and retention in the eye. The analysis was performed at the end of 1, 3, 5, and 7 days of formulations instillation. Left: regular fundus camera images of the eyes; right: fluorescent fundus camera images. Adapted with permission from [150], copyright (2012), American Chemical Society.

Yang et al. [150] reported the production of non-toxic polyamidoamine (PAMAM) dendrimer hydrogel/poly(lactic-co-glycolic acid) (PLGA) NP platform (HDNP) to treat glaucoma. The dendrimers were loaded with brimonidine and timolol maleat, which underwent an enhanced uptake together with a slow release in vitro up to 28–35 days. The topical administration of the same drug-loaded denndrimers in normotensive adult Dutch-belted male rabbits strongly reduced the introcular pressure (IOP) of about 18% maintaining a glaucoma reduction for four days. Conversely, Individual Dendrimer Hydrogel Formulations (DH) and NP Formulations (NP) decreased the disease symptoms only for 48 h (Figure 6).

### 5.4. Active and Self-Propelling Nanoparticles: New Generation of Nanobots in Retinal Disease

The customization of NPs formulation at a molecular level has interesting outcomes for reproduction of fundamental phenomena such as macro- and microorganisms’ survival mechanisms [151]. For example, bacteria, uni- or multicellular organisms, and sperm cells are able to sense and actively respond to external stimuli by the generation of movement. In particular, they convert an external energy sources (temperature, magnetic fields, adhesion forces, chemical gradients) into mechanical work that induce migration. This is typical in processes such as thermotaxis, magnetotaxis, haptotaxis, chemotaxis [152]. By taking inspiration from nature, we have also now a panel of NPs able to self-propel towards a gradient of nutrients and to mimic biological taxis [153]. However, the development of such NPs still has many challenges because at nanoscale level, the physical principles of propulsion are different due to the presence of the Brownian effect that can interfere with nanomptors by collisions with solvent molecules. One of these challenges is the generation of particles having asymmetrical geometries at the nanoscale, as this provides guidance for the movement directionality. Bacteria like *Escherichia coli*, in fact, achieve propulsion by non-time-reversible motion of long flagella, and this asymmetric shape of the body is critical for motion generation in a specific direction [154].

**Figure 7:**
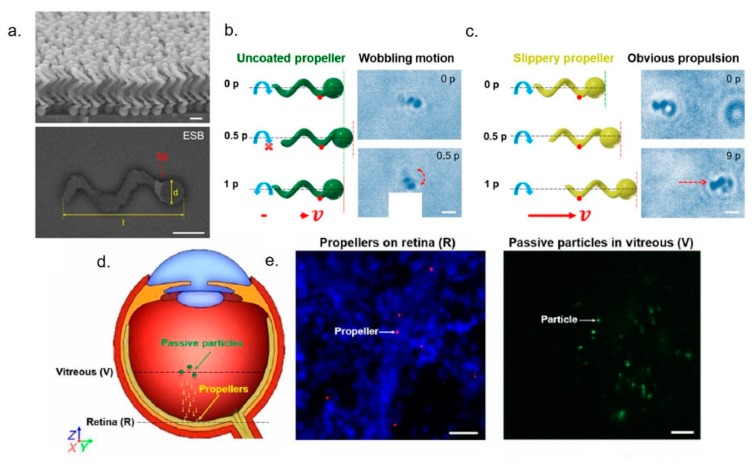
(**a**) SEM and ESB-SEM images of the micropropellers. (**b**,**c**) incomplete rotation of an uncoated micropropeller for one period (p) in the vitreous was showed in schematic and time-lapse microscopy, whereas the coated propeller exhibited propulsion under magnetic stimuli in vitreous for 1.5 s (nine periods). Red arrows showed the propulsion direction of propeller. The original position of nanotools and after the rotating magnetic field application were showed with green and red dashed lines. Scale bars, 1 µm. (**d**) Schematic illustration of micropropeller movement in the vitreous. (**e**) Left: fluorescent acquisitions showed the micropropellers labelled red on retina. Right: passive fluorescent particles were accumulated near center of vitreous. Nuclei of cells were stained with DAPI. Scale bar, 20 µm. Adapted from reference [155] under the terms of the Creative Commons Attribution-Non Commercial license.

It is clear that self-propelling NPs represent a new powerful tool in drug delivery, which is usually dominated by NPs passive diffusion. It should not come as a surprise that such strategies have been employed also for delivery in ocular medicine. For example, Wu et al. [155] designed micro-vehicles that can actively propel through the vitreous humor to enrich retina. The movement can be activated by helical magnetic micropropellers (~120 nm in diameter and ~400 nm in length) with liquid layer coating to reduce the adhesion of biopolymeric network. These nanovectors propelled in nanoporous hyaluronan solution, a fluid mimicking the vitreous. The inspiration for nanotool development is due to the liquid layer that characterizes carnivorous *Nepenthes* pitcher plant, which presents a slippery surface on the peristome to catch insects [156,157]. Porcine eyes were used as a model of human eyes: applying the external magnetic field, micropropellers exhibited controllable propulsion from eyeball to retina within 30 minutes. Instead, passive silica microparticles did not penetrate the vitreus (Figure 7). Nanorobots were also used to perform microsurgery on the retina and surrounding membranes thanks to the possibility of injecting them elsewhere in the body and bringing the drug to the target eye region [158]. Some intravitreal implants have recently developed to perform more clinical-inspired experiments. These nanotools, constituted by polymeric containers (silicon, poly(vinyl alcohol), or ethylene vinyl acetate), were placed in the posterior segment of the eye and they are permeable to lipophilic drugs. They did not display any toxicity and had a long retention time. These nanosystems have been extremely useful to treat proliferative diabetic retinopathy, retinal vascular occlusion, and uveitis [159].

## 6. Conclusions and Perspectives

It is established that eye damage is a leading cause of irreversible vision loss resulting from ocular diseases. The current standard treatments helped in both preventing and curing several pathological conditions, especially when related to the deep sections of the eye. However, many of these methods are rather invasive in terms of procedures, with a consequent possibility of promoting infection, inflammation, or even retinal detachment with potential risk of vision loss. These limitations impose considerable costs on patient quality of life and have an enormous economic impact on the health care system. The progress in the nano-engineering field can help boosting the discovery of a new approach for ocular diagnosis and therapy. Noble metals NPs and bio-inspired NPs represent a powerful tool to overcome all the limitations coming with standard treatments due to their physico-chemical properties. NPs can in fact penetrate deeper inside the ocular segments and also locally deliver high dosages of drugs, enriching local drug concentration when applied topically. In addition, the plasmonic properties of Au and Ag can have an added value to contrast eye diseases. However, numerous challenges still remain to be addressed as there are still limited numbers of in vivo studies. In addition, a deeper toxicology assessment is required for the development of new systems for ocular treatments.
